# Reducing the Future Risk of Trauma: On the Integration of Global Disaster Policy within Specific Health Domains and Established Fields of Practice

**DOI:** 10.3390/ijerph15091932

**Published:** 2018-09-05

**Authors:** Lennart Reifels

**Affiliations:** 1 Centre for Mental Health, Melbourne School of Population and Global Health, The University of Melbourne, Melbourne, VIC 3010, Australia; l.reifels@unimelb.edu.au; Tel.: +61-3-8344-0667; 2 Disaster Research Unit, Department of Political and Social Sciences, Free University of Berlin, 12165 Berlin, Germany

**Keywords:** disaster, disaster risk reduction, Sendai framework, mental health

## Abstract

The global increase in the frequency and severity of natural hazards and extreme climatic events necessitates more efficient global and national strategies to reduce the likelihood and impact of traumatic consequences for disaster-affected populations. The recent inclusion of mental health in the Sendai Framework for Disaster Risk Reduction marks a pivotal point in the recognition of the significant burden of disasters on mental health, and a global commitment to reducing its impacts. Nevertheless, effective agreement implementation and efforts to reduce disaster mental health risks are facing significant challenges. These include a lack of clarity about the conceptual interlinkages and place of disaster risk reduction principles within the field of disaster mental health, which is traditionally marked by a prevailing recovery orientation, and the need for effective translation into disaster mental health policy and practice. Therefore, this study drew on data from interviews with European disaster mental health and risk reduction experts in order to appraise the merit and implications of a global disaster risk reduction policy for advancing population mental health in the context of disaster. Study findings outline existing opportunities, challenges, and key strategies for the integration of disaster risk reduction within disaster mental health policy and practice.

## 1. Introduction

At the global level, human mental health and wellbeing is being increasingly jeopardised through rising trends in population exposure to severe natural hazards and extreme climatic events [1,2]. Despite the often remarkable resilience of communities to the onslaught of such events, decades of disaster mental health research have demonstrated the significant burden that mass-traumatic events can inflict on the mental health of affected populations [3]. This mental health impact can vary in intensity, course, and duration, and find expression in temporary distress reactions among a large majority, ongoing mental health problems of mild to moderate severity, as well as more severe mental health issues (such as post-traumatic stress disorder or depression) among a significant minority of those affected by disaster [4]. Apart from constituting an important outcome domain in its own right, population mental health also plays a key role in facilitating effective rebuilding and recovery efforts and in terms of broader social, socio-economic, and societal functioning [5].

International recognition of the need to better manage the varied risks associated with disaster exposure has resulted in seminal intergovernmental agreements and policy frameworks to guide global and societal disaster risk reduction [6,7]. The Sendai Framework for Disaster Risk Reduction 2015–2030, which was adopted by 187 United Nations (UN) member states in March 2015, reflects an important conceptual shift in global disaster policy—away from managing disaster impacts and consequences, toward a proactive approach to managing and reducing disaster risks. Although this relatively subtle shift in global disaster policy may be unbeknownst to many outside of the field, it is likely to have important practical ramifications for concerted societal efforts to address disaster risks across varied sectors and practice domains over the next 15 years. It further requires a thorough understanding of both existing and newly emerging disaster risks facing societies today and the adoption of proactive and effective strategies to manage and reduce these risks in future.

The Sendai Framework is structured around the key elements of a global goal and expected outcome, operationalised through seven global targets primarily aimed at reducing: disaster mortality, the number of people affected, economic losses, damage to infrastructure, and disruption of basic (e.g., health) services. In addition, four cross-cutting priority areas for global action highlight the critical importance of: (1) understanding disaster risk, (2) strengthening disaster risk governance, (3) investing in disaster risk reduction, and (4) enhancing disaster preparedness for effective response and to ‘build back better’. As part of a concerted implementation effort, the Sendai Framework also outlines local, national, and regional level stakeholder roles and responsibilities.

One of the cornerstones of the Sendai Framework is its strong emphasis on human health. This is particularly evident in 35 direct references to health, the explicit inclusion of health in the expected Sendai outcome (“the substantial reduction of disaster risk and losses in lives, livelihoods, and health and in the economic, physical, social, cultural, and environmental assets of persons, businesses, communities, and countries”), and direct links to health in four of the framework’s seven global targets [8,9]. Within the context of this strong health emphasis, the explicit inclusion of mental health in the Sendai Framework marks a pivotal point in the recognition of the significant burden of disasters on the mental health of affected populations, and a simultaneous global commitment to reducing its impacts [10]. Mental health features, perhaps most prominently, as a national and local level responsibility under Sendai’s priority action area (4) in terms of the aim to “enhance recovery schemes to provide psychosocial support and mental health services for all people in need” (paragraph 33o); whereas other key action areas underpin the critical importance of a better understanding of disaster risks across varied societal sectors and domains.

However, despite the formal inclusion and recognition of the importance of population mental health, significant challenges remain to the effective Sendai agreement implementation and associated efforts to reduce disaster mental health risks and consequences. These include a lack of clarity about the conceptual interlinkages and place of disaster risk reduction principles within the field of disaster mental health, which is traditionally marked by a prevailing recovery orientation, and the need for translation into disaster mental health policy and practice.

Conversely, key aspects relevant to disaster mental health have thus far received relatively little systematic attention in the context of disaster risk reduction frameworks. This, however, is in stark contrast to more general and public health domains, which are increasingly being considered in disaster risk reduction contexts [8,9,11,12]. As a direct consequence, the Sendai Framework currently provides little conceptual elaboration of the nature of disaster mental health risks or necessary guidance for future societal strategies to reduce and manage associated mental health risks and consequences of extreme climatic events and disasters. In view of the inevitable limits of global disaster treaties to provide health domain-specific guidance, there is therefore an urgent need to carefully consider the implications of the Sendai Framework for specific health domains, and to facilitate its integration within established fields of practice [13].

For brevity, this article adopts an inclusive definition of disaster mental health (DMH) as a scientific discipline and field of practice that is concerned with understanding, preventing, and addressing mental health problems and promoting psychosocial wellbeing and resilience in disaster and emergency contexts. Thus understood, DMH is not merely limited to efforts that seek to better understand or address psychiatric phenomena via therapeutic interventions, but rather includes recognition of the vital importance of a broader range of psycho-social factors, outcomes, and support strategies in disaster and emergency contexts. Emerging from traditions, such as trauma and stress studies, and incorporating related humanitarian practice fields, such as Mental Health and Psychosocial Support, DMH has amassed a considerable body of knowledge about the potential spectrum of disaster mental health outcomes and relevant intervention and support strategies over time [14,15]. Yet, traditionally, the primary focus of DMH strategies has been on facilitating recovery in the wake of disaster, whereas the field has remained fairly oblivious to broader developments in global disaster policy. In view of this circumstance, the current shift in disaster policy in the context of the Sendai Framework—from managing disaster impacts to reducing disaster risks—provides a pertinent and timely opportunity to examine the intersection of these two fields in more depth, and to explore the DMH field through a disaster risk reduction (DRR) lens.

This study therefore drew on data from interviews with European disaster mental health and risk reduction experts in order to appraise the merit and implications of global disaster risk reduction policy for advancing population mental health in the context of disaster. More specifically, this study examined the conceptual nature of disaster mental health risks, as well as concrete opportunities, current challenges, and key strategies for the integration of disaster risk reduction within disaster mental health policy and practice.

## 2. Materials and Methods

This study drew on data from interviews with 17 European key informants (including 14 DMH and 3 DRR experts), which were conducted between May and September 2016, as part of a larger research project that examined the early implementation of the Sendai Framework in Europe. In view of the relatively small pool of key informants in this area, this exploratory study adopted a purposive sampling frame informed by prior stakeholder mapping to recruit eminent DMH and DRR experts at the European regional and national levels (in the UK, the Netherlands, and Germany) for participation in semi-structured interviews. Participating DRR experts represented three of the 17 DRR experts recruited for the larger study who were both directly involved in the Sendai implementation and in a position to complete an interview schedule focused on disaster mental health. Key informants (10 men, 7 women) were typically affiliated with scientific institutions and national DMH or DRR advisory bodies across the UK, the Netherlands, and Germany, or European-based advisory bodies with a broader international mandate.

Semi-structured interviews were conducted on the basis of an interview schedule. The schedule included 15 questions that explored the intersection of DRR and DMH by eliciting information on: principal mental health risks in disaster contexts, DMH strategies to address these risks, and existing opportunities, current challenges, and key strategies for the integration of disaster risk reduction and disaster mental health. A copy of the full interview schedule is available from the author upon request. The interviews, which lasted between 40 and 95 minutes, were conducted either in person or remotely (via telephone or Skype), audio-recorded, and analysed thematically using NVivo11 (QSR International Pty Ltd., Melbourne, Australia). All key informants provided consent for study participation. The study protocol was approved by the Human Research Ethics Committee at the University of Melbourne (ID: 1646655.1).

## 3. Results

Key themes resulting from the expert interviews are summarised in the following, with the study’s thematic coding framework and illustrative participant quotes outlined in Table S1.

### 3.1. Principal Disaster Mental Health Risks

Key informants were asked about what they would regard as the principal mental health risks in disaster contexts. The principal mental health risks identified by the experts fell into five broad thematic categories. The first two of these highlighted commonly established risk factors for adverse mental health outcomes, as well as newly emerging risks that were contextually bound to the unfolding European refugee/migration crisis. The three remaining categories were focused on risks associated with the conduct of appropriate disaster mental health responses and intervention strategies, e.g., in relation to the adequate recognition and detection of complex mental health impacts, the provision of appropriate mental health support, and overarching disaster response communication and coordination.

#### 3.1.1. Common Risk Factors

Key informants frequently identified established risk factors for adverse mental health outcomes. These factors included, amongst others, the nature of the hazard, exposure level, peri-traumatic and post-event reactions, various forms of loss (of persons, resources, livelihoods), lack of (received or perceived) social support, distress impacting on normal functioning, the decay of the recovery environment, and secondary stressors in the disaster aftermath (related to damaged infrastructure, lacking health support, social conflict, and uncertainties about the future). Vulnerable population groups identified to be at particular risk and warranting special attention included those with pre-existing difficulties or severe mental illness, children, the elderly, socially isolated individuals, and people with disabilities.

#### 3.1.2. Emerging Risks (European Migration Crisis)

A second set of emerging mental health risks revolved around the unfolding refugee and migration crisis in Europe, with many experts indicating involvement in this crisis either inside or outside of Europe. Principal mental health risks identified in this context were often reflective of underpinning social and physical determinants of health. These highlighted the role of displacement as a major mental health risk factor given the loss of continuity of care, the impact of family separation (on anxiety, depression, identity loss), as well as frequent occurrences of unresolved missing person situations (which provided no opportunity for closure or appropriate mourning). Mental health risks specific to younger generations were posed by: disrupted education and development paths for youth (during an already challenging and critical developmental stage); the impact of disrupted schooling and language barriers on cognitive development; as well as lacking access to normal peers, play, and structure for children; and the impacts of malnutrition on the mother-child bond, routine lactation, and cognitive/physical development of infants.

#### 3.1.3. Recognition of Complex Mental Health Impacts

Failure to recognise the complexity and diversity of mental health impacts in disaster-affected populations was identified as a pivotal risk in the design and conduct of appropriate disaster mental health responses. Importantly, this included the risk of failing to detect those individuals who proceed to develop more persistent or severe mental health problems, distinct from those who initially experience common transitory distress responses. The shortcomings of a reductionist view were highlighted in terms of the failure to differentiate varying mental health risks of specific population groups (e.g., those affected directly or indirectly, or first responders), and in terms of a sole focus on specific signature disorders (such as post-traumatic stress disorder), rather than the relevant broader spectrum of disaster mental health outcomes and psychosocial consequences. Over-pathologisation on the part of disaster response planners further entails the risk of mismanaging normal human distress reactions to abnormal or disastrous circumstances. However, under-pathologisation may run the risk of failing to identify or reach those with significant mental health problems who may not otherwise readily access or seek support. Varied mental health risks posed by different disaster types and contexts, and interactions with pre-existing problems in disaster-affected communities, further compound the challenge of recognising complex disaster impacts in the design of appropriate mental health responses.

#### 3.1.4. Provision of Appropriate Mental Health Support

Specific risks associated with the provision of appropriate mental health support to disaster-affected populations include the potential use of ineffective early interventions (such as debriefing), the lack of timely psychosocial care, the absence of clear and timely community information, and the lack of recognition of longer-term mental health provisions that needed to be put in place. In view of diverse emerging mental health problems and known barriers in the access to mental healthcare, the need was highlighted for a delicate balance between an emphasis on proactive support intervention and a stance of watchful waiting. This balance would help optimally align support efforts with the stage of recovery and nature of presenting problems, and not alienate affected individuals and communities in the process.

#### 3.1.5. Disaster Response Communication and Coordination

Broader mental health risks were also identified in the context of disaster response communication and coordination. Non-consultative and top-down approaches of disaster response authorities can create mental health risks for affected communities further down the track. Politician behavior in disaster contexts can also be informed by and affecting public sentiment, whereas public perception of the way in which presenting health problems are handled or addressed by responsible authorities may pose a political risk for decision makers. Inherent risks of public disaster response communication included a general lack of communication, the uncertainty of (intended) outcomes, and possibility of unintended consequences. Moreover, disaster-poor countries can face challenges in communicating disaster risks to the wider public with a view to motivate self-protective action, which may lead to a stronger reliance on public authorities and experts in disaster contexts. Hierarchically organised civil protection systems, which are not traditionally citizen-centric, have therefore often failed to recognise existing community agency, resources, and self-help potential, or have struggled to translate a prevailing rhetoric of citizen engagement into effective practice. Conversely, the lack of public risk awareness, proper citizen engagement, and overarching stakeholder coordination and cooperation also increases the risk of adverse disaster outcomes and consequences.

### 3.2. Current DMH Strategies to Address Risks

When asked about key strategies through which the DMH field was addressing these principal disaster mental health risks, experts identified strategies within four main areas.

#### 3.2.1. Development of Guidelines and Response Plans

The development of overarching psychosocial disaster guidelines and response plans was seen as pivotal to the design and conduct of effective disaster responses and to addressing mental health risks in disaster contexts. Specifically, organisations with routine involvement in disaster responses will need to have psychosocial disaster plans in place. Although generic plans may never be specific enough to capture unique disaster circumstances, these nevertheless provide an important planning framework that can be adapted to concrete scenarios.

#### 3.2.2. Mechanisms to Identify and Direct People to Appropriate Support

Key mechanisms to detect and direct disaster-affected individuals to appropriate mental health support included the establishment of on-scene information and advice centres, the implementation of psychological screen and treat programs, and the establishment of disaster health registers. The role of public mental health awareness and wellbeing campaigns was raised in this context, as was the newly-developed concept of health passports for refugees and migrants, which may assist in providing greater continuity of care and access to vital medications across national boundaries.

#### 3.2.3. Psychosocial and Mental Health Support Strategies

Designated mental health support strategies emphasised the need to provide screening, effective early intervention, and psychosocial support within the broader context of practical assistance. The need for basic psychosocial support strategies that could be provided by lay people and volunteer-based disaster relief agencies was highlighted. Specific psychosocial support strategies identified included the creation of safe spaces for vulnerable groups (in terms of child-friendly spaces, non-formal schooling, or breast-feeding tents), as well as the provision of parental support, livelihood skill building, general information, or legal advice. Awareness raising for the specific situation and issues affecting (and advocating the protection of) vulnerable groups was considered particularly critical in the context of violence against women and for residents of psychiatric institutions. The integration of mental health support within primary care and ceasing of opportunities to strengthen existing mental health systems and ‘build back better’ after disaster were equally highlighted as key avenues to address systemic mental health risks in disaster contexts.

#### 3.2.4. Disaster Preparedness Planning

Other strategies and avenues to reduce disaster mental health risks were identified in the context of disaster preparedness planning. These included the provision of dedicated first responder training, the establishment of organisational support systems, mechanisms to facilitate multiagency networking, cooperation and joint planning, and the mapping of existing services. The importance of prior vulnerability and needs assessments (including geographical mapping of vulnerable groups) was highlighted, as was the need to involve these groups and the broader public in disaster preparedness planning. The resulting need to adapt existing alert systems, evacuation plans, and risk/crisis communication to the needs of vulnerable groups was emphasised, as was the potential to adopt a stronger focus on resilience-building activities throughout the whole cycle of disaster prevention, preparedness, response, and recovery planning.

### 3.3. Challenges to DMH/DRR Integration

Several questions explored the intersection of DMH and DRR in more depth, including existing challenges and opportunities for the integration of the two fields. Key integration challenges highlighted DMH and DRR as rather separate fields of practice, marked by practitioners from different professional backgrounds and a mutual lack of awareness. The use of technical jargon on either side was seen as an impediment to effective integration, as was the lack of DMH stakeholder involvement in the Sendai implementation process. The relative vagueness of the Sendai Framework, which does not specify key mental health risks to be addressed, along with its broad aims were seen to harbor the potential to lead to mental health initiatives that are not necessarily the most beneficial. Key challenges to greater recognition and use of existing DMH strategies within DRR included the lack of an empirical evidence base for specific psychosocial support strategies (which science requires to inform policy), and challenges in effective targeting of more preventative resilience-building initiatives before disaster strikes. Conversely, it was recognised that the Sendai implementation process did not necessarily follow a uniform international pattern but rather required unique integration within national systems and structures. Potential professional role and resource implications of the integration of the two fields were raised in terms of the necessary willingness of professionals to step outside of their brief, the perception that extra work or resources may be required, or that a stronger focus on community empowerment within DMH could result in less funding for traditional clinical or other core work. Other integration challenges included the limited sustainability of project-based initiatives, and lacking recognition of the vital role of DMH stakeholders in joint pre-disaster planning (beyond common ad-hoc involvement in response and recovery).

### 3.4. DMH/DRR Integration Opportunities

Notwithstanding these challenges, experts identified wide ranging opportunities for the stronger integration of the two fields. Key integration avenues involved the use of inter-sectoral platforms, joint stakeholder meetings, and preparedness summits, as well as disaster-related information and referral websites, and mobile phone applications. Joint DRR-DMH fact sheets and DRR primers for DMH staff may facilitate mutual practitioner understanding. Building on existing European projects and networks, the development of a shared European position was regarded as useful. In this context, major disaster events were also seen as progress catalysts that can provide opportunities to explore new solutions and trial different ways of operating.

DMH experts and practitioners can facilitate DMH/DRR integration by advocating the recognition of mental health issues in disaster prevention and response planning, integrating mental health knowledge at higher strategy and local planning levels, providing guidance on where mental health funding and efforts are best spent, and by continuing to build the underpinning DMH evidence base. Simultaneously, it was recognised that DMH must become better at providing clear and understandable messages and at marketing DMH knowledge, so that it can be more readily used by local authorities and stakeholders. Other DMH contributions to foster integration included psychosocial capacity analysis at the municipal level, prior vulnerability mapping, and knowledge and skill building in vulnerable groups to enhance self-help capacity and psychosocial stress management.

Integration can also be facilitated by expanding the prevailing DRR mandate to include mental health, such as by addressing psychological stressors in public crisis communication, including mental health messaging in non-formal education, as well as through professional selection and equipment of first responders with the vital skills to handle common stress reactions.

Promising conceptual interlinkages that facilitate future DMH/DRR integration efforts revolved around a shared focus on resilience building, the use of health promotion strategies, community-based and self-help approaches, and community mobilisation concepts that are already embedded in international guidelines for psychosocial emergency response. Other interlinkages exist between health and disaster literacy approaches, and between healthy cities and urban resilience frameworks.

Formal integration steps highlighted the benefits of incorporating DMH guidelines as an appendix to the Sendai Framework, the need to operationalise DMH/DRR linkages and interventions to support broader application and effectiveness testing, devote project funding to integration efforts, and mandate stronger interdisciplinary and interagency work, such as in joint disaster planning.

## 4. Discussion

The findings of this exploratory study provide a first look at the DMH field through a DRR lens, and a vital perspective on the intersection of these two fields that can facilitate future integration efforts. Study findings indicate that it is clearly early days in terms of the formal integration of the fields, and that Sendai disaster policy awareness within the mental health field may be limited. Yet, the impending application of DRR concepts and approaches across varied societal sectors and health domains in the context of the Sendai implementation provides a pertinent and timely opportunity to examine its conceptual and practical implications for DMH and other established fields of practice.

### 4.1. Conceptualising DMH Risks: The Place of Risk Within DMH

Application of the Sendai objective of understanding disaster risk within the context of a specific health domain and field of practice (such as DMH) inevitably involves the task of fleshing out an otherwise generic (or content-neutral) notion of disaster risk, with domain-specifically relevant content that is consistent with the broader Sendai notion. A better understanding of pre-existing notions of disaster risk held among practitioners within a given health domain therefore constitutes an important preliminary step in developing a shared conceptual understanding and joint future integration and work agenda.

In appraising the nature of principal DMH risks identified by the experts, and the place of risk within the DMH field more broadly, it is evident that DMH has developed an existing risk vocabulary (and associated evidence base) that is primarily centered on established risk factors for adverse disaster mental health outcomes. Such risk factors include a wide range of predisposing factors (such as age, gender, socio-economic status, or pre-existing difficulties), as well as factors related to disaster exposure and secondary stressors, all of which have been well established in the literature [3].

Nevertheless, beyond the primacy of this risk factor-driven understanding, the broad scope of identified principal DMH risks also spanned newly emerging mental health risks (contextually bound to the European migration crisis) and risks associated with the design and delivery of adequate support interventions (such as the recognition of complex disaster mental health impacts, the provision of appropriate mental health support, and broader disaster response communication and coordination). As a whole, these principal DMH risks are reflective of the developmental stage, existing sophistication, and key challenges of the field. Moreover, all these DMH risk notions (either directly or indirectly) have a shared concern with the potential for adverse disaster mental health outcomes.

Specific DMH risk factors identified in this study are structurally different from the broader (probabilistic) notion of disaster risk, elaborated and defined in the Sendai Framework as “The potential loss of life, injury, or destroyed or damaged assets which could occur to a system, society, or a community in a specific period of time, determined probabilistically as a function of hazard, exposure, vulnerability, and capacity” [16]. DMH risk factors are perhaps more akin to various elements of this definition, such as the key concepts of vulnerability, exposure, and (as in the case of the latter DMH risks) capacity.

When DMH risks are classified in terms of the specific nature of adverse disaster outcomes considered, it becomes possible to distinguish at least three different types of DMH risk, and to conceive of DMH risk more generally, as the likelihood and severity of:

(1) Newly emerging or exacerbated mental health issues among disaster-affected populations (Type A)
(2) Adverse impacts on existing mental health support systems (Type B), for example, in terms of client/staff safety and wellbeing, infrastructure damage, service disruption, surge capacity, evacuation, and business continuity
(3) Secondary effects of A and B on individual, community, business, and societal functioning (Type C)

Beyond these principal DMH risk types, it is also possible to identify more intermediate DMH risks, such as:

(4) Lack of DMH preparedness or capacity (Type D)
(5) Poor quality, lacking efficiency, efficacy, and coordination of DMH responses (Type E)

Nevertheless, although these latter intermediate risks may constitute ongoing key challenges of the field, these can also generally be regarded subordinate to principal DMH risks of Types A and B. In considering the nature of domain-specific DMH risks of Types A–C, it is again helpful to draw upon the broader Sendai notion of disaster risk. This notion calls us to move beyond the mere consideration of adverse outcomes, and to adopt a comprehensive understanding of disaster risk as determined by the key aspects of hazard, exposure, and existing vulnerabilities and capacities [16].

### 4.2. Current Focus of DMH (Risk Reduction) Strategies

In view of the nature of identified mental health risks in disaster contexts, much of the existing arsenal of DMH strategies has traditionally been focused on addressing these risks during disaster response and recovery phases. As such, these strategies could be seen to require the prior occurrence of disaster event exposure in order to become relevant or applicable. Yet, interview data also suggested an increasing trend toward the exploration of more proactive, preventative, and resilience building initiatives within DMH, which, while still fledging in terms of an empirical evidence base [17], would also be well aligned with broader developments in disaster policy and the move toward the promotion of disaster resilient communities, health systems, and societies [18,19].

### 4.3. Sendai’s Dual Challenge for DMH

In this context, it is interesting to note that the principal reference to mental health in the Sendai Framework (Priority 4, paragraph 33o; see Introduction) encapsulates an implicit understanding of DMH that firmly locates the role of the field within disaster recovery and building back better phases. As such, this conceptual understanding also reinforces the traditional DMH focus and self-understanding. It is also in this narrow sense that the genuine conceptual challenge posed by the Sendai Framework for DMH policy may be considered comparatively negligible, notwithstanding substantial variability in the existing capacity of countries to effectively apply DMH policy and know-how in practice [20]. Put differently, the implications of this narrowly conceived Sendai challenge for DMH may be less about the aim of finding new and innovative ways of doing things, but rather about becoming more professional at those activities in which the field at large is already engaged; perhaps resulting in a greater need for capacity building or upscaling (particularly in resource-poor countries), rather than broader reorientation or reinvention.

Yet, while undoubtedly more work must be done in terms of strengthening the existing capacity, effectiveness, integration, and sustainability of DMH systems [21], Sendai’s conceptual challenge for DMH policy and practice (in a broader sense) goes further. When Sendai’s underpinning philosophy of understanding and reducing disaster risk is taken to heart, it becomes possible to look beyond DMH’s established role as post-disaster savior, and to explore the potential role that the DMH field (via existing or future strategies) may be able to assume in reducing disaster risks and in averting adverse disaster mental health consequences, before these occur. The implications of this broader Sendai challenge for DMH may thus require a potential shift in conceptual emphasis—away from the current focus on downstream interventions and toward the exploration of more proactive upstream strategies that are effective at reducing DMH risks [13].

### 4.4. Sendai Implementation Requirements

Global Sendai Framework implementation between 2015 and 2030 will be monitored on the basis of national progress reporting across UN member states against agreed targets and indicators [16]. The ability of member states to demonstrate progress in terms of not only the existence but outcomes of effective DRR strategies has direct implications for societal sectors and health domains, and for the field of DMH. Key Sendai progress indicators of relevance to health include quantifiable reductions in the number of deaths (A-2), missing persons (A-3), injured or ill people (B-2), each per 100,000 population; as well as in the number of destroyed or damaged health facilities (D-2), and disruptions to health services (D-7) attributable to disasters [16,22].

When these health-related Sendai implementation requirements are viewed through the lens of the above DMH risk typology, these would primarily call for a better understanding and the reduction of principal DMH risks of Type A (in alignment with Sendai indicator B-2) and Type B (in alignment with indicators D-2 and D-7), whereas wider societal benefits of monitoring, enumerating, and reducing secondary, or more intermediate DMH risks of Types C–E, are also evident.

In this context, the Bangkok Principles, in support of the implementation of the health aspects of the Sendai Framework [23] and related UN guidance directives, also specifically highlight the importance of incorporating relevant (mental) health indicator data within comprehensive national disaster risk assessments [24], associated disaster loss databases [25], and multi-hazard early warning systems. Future monitoring of Sendai implementation progress in terms of the reduction of principal DMH risks is therefore critically dependent on the systematic identification, collection, and incorporation of mental health indicator data within these planning and reporting processes to ascertain reduced disaster impacts at population and service system levels over time.

### 4.5. Study Limitations

As the first study explicitly focussing on the nexus of DMH and DRR, this study adopted an open-exploratory and not theoretically-driven approach to examining DMH risks, strategies, and conceptual interlinkages of these fields from the perspective of European DMH and DRR experts. In view of the social construction of disaster risk [26], it is conceivable that exploration of these issues with other stakeholder groups (such as lay people or professionals from other regions) may have resulted in different conceptualisations of risk or priority strategies to address these. Specific DMH risks identified in this study may also, at least in part, be socio-historically contingent upon major events unfolding in the study region at the time. Although interview participants in this exploratory study represented eminent DMH and DRR experts at each of the targeted European regional and national levels, the purposive study sampling frame and relatively small sample size may have limited the representativeness and generalisability of study findings. The broader project policy focus on the Sendai Framework implementation across Europe placed restrictions on the extent to which its implications could be examined for more specific national or local DMH practice traditions.

### 4.6. Implications and Future Directions

DMH is in a favorable position to tackle the Sendai challenge to both inform DRR, and in turn, be informed by DRR. Expert identification of mental health risks and strategies does not necessarily imply that these risks are generally well understood or that such strategies are already widely in place across countries. The inclusion of mental health in the Sendai Framework is laudable, yet its general formulation does not reflect the existing key challenges and sophistication of the DMH field. DMH therefore needs to guide DRR on the relevant mental health risks and aspects of disasters and on the choice of effective strategies to address these. In turn, a shift in DMH orientation and further steps will be required to effectively translate DRR principles and concepts into DMH policy and practice.

This study outlined several strategies and conceptual interlinkages that can facilitate future DMH/DRR integration efforts and provided valuable insights and lessons for the integration of global disaster policy in other health domains and established fields of practice.

Future efforts to integrate global DRR policy within a given health domain need to address several interrelated levels. At a conceptual level, key DRR concepts and principles, such as disaster risk and risk reduction, need to be examined in terms of their compatibility with and implications for prevailing doctrines and practice approaches [27]. The application of a DRR approach within a given health domain, such as DMH, thus first and foremost requires completing a rather abstract notion of disaster risk with health domain-specifically relevant content and meaning. Whereas the Sendai Framework provides broad parameters and agreed-upon definitions of key DRR concepts, it is essential that we turn to practitioners and experts within these fields to better understand principal disaster risks and existing strengths and limitations of key strategies to address these risks at present.

At a policy level, integration can be facilitated through systematic mapping of the Sendai Framework against existing policies and practice guidelines to identify key areas of overlap, existing gaps, inform strategic future planning, and focus areas for further development. Insightful examples of comprehensive Sendai mapping exercises already exist in regard to EU policies [28], and the U.K.’s existing doctrine of integrated emergency management [29]. Future efforts to integrate DRR within DMH would thus benefit from systematically mapping the Sendai Framework against DMH policies and guidelines at varied entity levels including international, national, sub-national, and agency levels.

At a translational level, results of these foundational exercises can inform the shape and focus of key activities to operationalize existing linkages between these fields. These include the identification of promising bridging approaches or innovative case studies—at policy, service, or practical intervention levels—that can be applied more broadly and tested for effectiveness. Capitalizing upon and further developing existing avenues for joint cooperation between DMH and DRR sectors will become increasingly important, as is ensuring adequate DMH and health sector representation at all levels of the Sendai implementation process. In this respect, it is critical that mental health specific indicators be developed that align with broader Sendai indicators to facilitate ongoing monitoring and reporting of Sendai progress in terms of reduced mental health impacts at population and service system levels over time. One of the key strengths of the Sendai Framework is that it combines an ambitious and timely global policy program with broad multinational buy-in and clear guidance to facilitate its implementation at global, regional, national, and local levels.

Health domain-specific DRR integration efforts can complement broad-based, whole-society DRR approaches by highlighting the relevance of DRR policy and implications of key DRR concepts, addressing existing translation barriers, and helping to unlock the unique DRR potential within established fields of practice. Notably, designated DMH interventions constitute one key element in the broader arsenal of DRR strategies that can reduce future risk of disaster exposure, associated traumatic impacts, and adverse mental health consequences. Therefore, effective disaster mental health risk reduction requires the application and integration of effective DMH strategies in the context of broad-based DRR strategies [13]. To further strengthen its unique role and contribution in the Sendai implementation process, the DMH field needs to intensify its efforts to establish a sound evidence base to support the choice of effective interventions, to sustainably build its disaster preparedness and response capacity, and to actively explore the merit of more preventative upstream strategies. The recently established World Health Organization (WHO) Thematic Platform for Health Emergency and Disaster Risk Management Research [30,31] provides a promising overarching context in which to further examine principal disaster mental health risks (Types A–E) and more effective strategies to reduce these risks in future.

## 5. Conclusions

Successful Sendai Framework implementation and efforts to reduce disaster mental health risks critically hinge upon the application of both concerted broad-based DRR and effective DMH strategies, and on the effective integration of DRR principles within existing health domains and established fields of practice.

## Supplementary Materials

The following are available online at http://www.mdpi.com/1660-4601/15/9/1932/s1, Table S1: Thematic Coding Framework.
