# Supplementary material for: Reducing the Future Risk of Trauma: On the Integration of Global Disaster Policy within Specific Health Domains and Established Fields of Practice
Source: Int J Environ Res Public Health. 2018 Sep 5;15(9):1932. doi: 10.3390/ijerph15091932 (PMC6164068; doi:10.3390/ijerph15091932)
Supplement: Supplementary file 1 [file ijerph-15-01932-s001.pdf]

Table S1. Thematic Coding Framework.

Interview Domain (A priory code)	1st Level Theme (Semantic)	2nd Level Theme (Semantic)	Exemplary Participant Quotes Illustrating Key Themes
Principal DMH Risks	Common Risk Factors	Nature of exposure to hazard/extreme stressor<br>Peri-traumatic and post-event reactions<br>Loss (persons, resources, livelihood)<br>Lack of (received and perceived) social support<br>Distress impacting on functioning<br>Vulnerable groups:<br>- People with pre-existing difficulties<br>- People with severe mental illness<br>- Elderly<br>- Children<br>- Isolated individuals<br>- People with disabilities<br>Decay of recovery environment<br>Secondary stressors and victimisation<br>Uncertainties about future<br>Survivor guilt	*"the first thing is clearly the exposure to the risk, the primary stressor, either directly or indirectly, and then you've got the dose effect, the more you are engaged, the risk increases by your exposure to it."* (DMH 02)<br><br>*"I think exposure is the most important predictor in the development of psychopathology, exposure should be part of the risk"* (DMH 03)<br><br>*"the main risks are connected to the multiple losses of people, like loss of resources, loss of persons"* (DMH 12)<br><br>*"the level of support you get from your family and people around you is critical to the risks you run. Social support, the emotional, informational and pragmatic instrumental support. We have primary evidence that lack of provision of that, and what you expect, and what you receive, have different, have massive implications."* (DMH 02)<br><br>*"in terms of mental health, distress impacting on functioning is a very high-level risk early on"* (DMH 09)<br><br>*"the risk for vulnerable groups, for older people, for people with disabilities, for refugees, for specific vulnerable groups"* (DMH 12)<br><br>*"obviously people with pre-existing difficulties are more vulnerable than people without, and oftentimes you get a magnification of pre-existing difficulties"* (DMH 09)<br><br>*"the negative impact of the disaster on the recovery environment for mental health"* (DMH 13)<br><br>*"secondary victimisation, for example through media portrayals, inadequate compensation schemes, or legal procedures"* (DMH 11)
	Emerging Risks (European Migration Crisis)	Displacement<br>Loss of continuity of care<br>Family separation > anxiety, depression, identity loss<br>Missing people > no closure or appropriate mourning<br>Youth - disrupted education and development path<br>Children - disrupted schooling, language barriers, no access to normal peers, play, structure can impact cognitive development	*"It's so dangerous to your mental health, being displaced is a very major risk factor for several reasons, not the least of which is that you're often lost to follow-up, because somebody moves to somewhere else. Continuity of care and that's not just mental health care but the whole of your health care can be lost in the course of that. And there's quite a lot of work that shows how impactful that is."* (DMH 02)<br><br>*"The risks that we see are mainly related to families being separated, either in flight or because of the conscious decision to separate in the case of migration. And that often*

	Infants - malnutrition affects cognitive/physical development, mother-child bond, lactation	*leads to a lot of anxiety, depression, and sometimes a loss of identity."* (DMH 14)<br><br>*"For children particularly, if they have had disruptive schooling, or are unable to go to school, for whatever reason, it can be also because of the language, for instance a Syrian refugee who doesn't speak German, then it can affect their cognitive development, longer term, because they don't have access to their normal peers, normal play, and structure, within the class room environment as well"* (DMH 14)
Recognition of Complex Mental Health Impacts	Failure to detect people with persistent or severe mental health problems<br>Identification of mental health risks for different population groups<br>Failure to recognise complexity of mental health impacts (reductionist view)<br>Sole focus on PTSD (not broader spectrum of mental health, wellbeing and social consequences)<br>Mismanagement of normal reactions<br>Over-pathologisation / under-pathologisation<br>Mental health risks vary from disaster to disaster (interact with existing problems)	*"I think the main risk is to do with the failure to detect people who have persistent mental health problems and who are not going to respond to immediate short-term intervention. All the evidence seems to be that people with these more persistent problems rarely either seek or get appropriate treatment. That for me has always been the sort of central risk that needs managing, the risk of not identifying them in the first place, not getting appropriate psychological support services to them."* (DMH 01)<br><br>*"The risk, well to me, it's even the identification of it, not only for the individuals, but also for the responders, because they are just as much at risk."* (DRR 01)<br><br>*"it's quite complex, and more complex than perhaps people think. There's often people trying to take too much of a reductionist model about it, when planning how to deal with it, and that's definitely a risk when it comes to authorities taking it seriously and recognising what provisions need to be put in place in the longer term"* (DMH 09)<br><br>*"Maybe the biggest risk is that we always want to go for not losing anybody, and that we are afraid as a government or institution that we might miss somebody who develops PTSD - instead of focussing on what's best for the largest proportion."* (DMH 08)<br><br>*"being able to deal with the variety of different normal reactions that people have, I think is a key issue, so mismanagement of normal reactions"* (DMH 09)<br><br>*"From my experience from many disasters, these are very different. The El Al disaster gave a lot of unexplained medical symptoms, like rashes on the skin, but also sleep problems, PTSD and on the community level lots of conspiracy theories and paranoia. … We had a new year's café fire in a traditional fishing village with many burned teenagers. Here the already enormous abuse of alcohol and drugs in this village was the level to start with complications from burn seeing these youngsters all the time in the village. Here also PTSD was common. It is good to realize disasters happens in lives, so it burdens upon other problems already within a special context"* (DMH 06)

Provision of Appropriate Mental Health Support	What to do wrong/right after disaster<br>Proactive intervention vs watchful waiting<br>Use of ineffective early interventions<br>Lack of timely and clear/honest information<br>Lack of timely psychosocial care<br>Lacking recognition of long-term provisions	*"you can do too little or too much, you can be too inefficient as a psychologist about the people who are affected, which we know isn't the way to go, but on the other side there are always people you miss, so there's always the trouble of trying to find the right balance. This shows how difficult it really is to know how much you should be forward in your actions or rather wait until you get questions from people asking for help."* (DMH 05)<br><br>*"The lack of timely and clear/honest information and psychosocial care for victims. This is not always easy to achieve."* (DMH 05)
Disaster Response Communication and Coordination	Non-consultative, top-down approaches<br>Politician behaviour and public sentiment<br>Public risk communication<br>Public perception as political risk for decision makers<br>Erroneous assumption that all risk can be eliminated<br>Risk communication in disaster-poor countries<br>Lacking community disaster risk awareness and first responder preparedness<br>Lack of overarching disaster response coordination and interagency cooperation<br>Civil protection systems failing to recognise or mobilise community agency and self-help potential	*"There are other issues that affect your mental health, like how other people behave, how the rescue organisations behave, and that goes from the high level to the practical frontline. At the high level, if you institute top-down plans, so you arrive say as the police commander and you institute what you've been taught to institute, without consulting the local people, you are riding for a painful fall, which will make people's mental health worse. So, you've got to be very careful of 'top-downism'."* (DMH 02)<br><br>*"it's the interaction, sometimes you see that the prime minister says every single victim will be ok, and we know not everyone will be ok, and so sometimes they are not recognising what's really going on for people"* (DMH 06)<br><br>*"communication can also be a risk, because you can never be certain that you realised the result that you wanted to have"* (DMH 03)<br><br>*"we are a disaster-poor country, where the disaster topic is not very popular. So, in terms of risk communication, we have real difficulty communicating disaster risks, because people are not very open to it."* (DMH 10)<br><br>*"in Europe you often have a very good intervention team and strategy for the very acute phase and then you have a rather good health system, but between the acute phase and the health system the link is often missing, and this gap has to be closed"* (DMH 12)<br><br>*"It's the one thing over the years we got wrong every time. The task of how we get people to coordinate, and almost any activity across agency boundaries is very difficult. So how to extend that cooperation and engagement, I think that's critical for people's mental health, is having this kind of web of engagement."* (DMH 02)<br><br>*"We have a very hierarchically organised crisis management and civil protection system, which does not sufficiently recognise and embrace the existing competencies, creativity and self-help potential of citizens."* (DMH 10)

DMH Strategies to Address these Risks	Development of Guidelines and Response Plans	Overarching psychosocial support guidelines<br>Organisations having existing plans in place	*"Another key strategy is to develop psychosocial support plans beforehand"* (DMH 12)<br><br>*"I think the key strategies are, organisation, so trying to have existing plans in place, with people already trained up to a degree so that they can deliver them. No off the shelf plan is going to be bespoke enough for an individual disaster, but having a planning framework in place with principles to follow is important"* (DMH 09)
	Mechanisms to Identify and Direct People to Appropriate Support	Information and advice centres<br>Screen and treat programs<br>Disaster health register<br>Health passports (for refugees, migrants)<br>Public mental health awareness and wellbeing campaigns	*"There is the acute phase with the [regional medical disaster assistance organisation] and the information and advice centre"* (DMH 06)<br><br>*"you have to put in place something that will enable you to identify the people and then to follow them up and screen them and direct them towards appropriate treatments if they need it. … one initiative which came out of our working in the London Bombings was a disaster health register"* (DMH 01)<br><br>*"the Department of Health have mounted a screen and treat program for victims of this terrorist attack and the idea is that this will be available to victims of subsequent attacks"* (DMH 01)<br><br>*"the health passports, the way people have gotten around to thinking about it at the moment is, if you are a psychiatrist and you prescribe medication to someone, a migrant that you know is on the move to somewhere else in Europe, is to give them a piece of paper with the prescription. And to make sure the drug name is a generic name, and the drug is available within European countries."* (DMH 14)<br><br>*"formulate the right public health communication strategies to improve communication in disaster contexts"* (DMH 03)<br><br>*"every country needs something like an 'Are you alright?' campaign"* (DRR 01)
	Psychosocial and Mental Health Support Strategies	Mental health interventions in the context of broader practical and social support strategies<br>Screening<br>Effective early intervention<br>Basic psychosocial strategies (applicable by anyone)<br>Volunteer-based agencies' role in psychosocial support<br>Creation of safe spaces for vulnerable groups<br>Advocating protection (to prevent violence)<br>Attention to people in psychiatric institutions<br>Integration of mental health in primary care<br>Strengthening existing mental health systems	*"looking much more at psychosocial elements, and at psychological, psychiatric interventions in the context of broader social interventions, which are likely to be very powerful. It's going back to your hierarchy of needs, shelter, safety, money, etc and all of these things are critical and need to be done in tandem or even before psychological or psychiatric interventions."* (DMH 09)<br><br>*"There is some work in terms of accurately predicting who is going to develop difficulties, so screening sort of work, and then there's better work really on picking up individuals with difficulties and providing effective early interventions"* (DMH 09)<br><br>*"Psychological First Aid, I've got major concerns that, done wrongly, that could*

		become another intervention, and sort of the property of mental health professionals, which I don't think it should be. I don't think that's where it fits in, you don't want to make it too complex" (DMH 09)<br><br>*"more broadly, there's a lot of creation of safe spaces set up in emergency situations, and those take a variety of different forms, such as safe spaces for women and girls, if there is a high risk of SGBV. And I've also seen safe spaces for adolescents. They tend to be looking at non-formal schooling side of psychosocial support."* (DMH 14)<br><br>*"The natural disaster may be over, but sexual violence against women may be up. So, the protection response is really important to prevent exposure to extreme stressors. It's thinking about the determinants of mental health, in this case violence. As a mental health field, we should advocate for that. The second part is bringing in psychological skills to deal with the results of these stressors, so we are focussing on developing a manual for psychological interventions that reduce those psychological symptoms"* (DMH 13)<br><br>*"for people with pre-existing disorders one strategy is integration of mental health in primary healthcare, which we do through our MH Gap program. Another is specific attention to people in institutions, because people in institutions are extremely vulnerable. And there is an action sheet in the IASC guidelines. Then, in general, discussing the issues, in form of advocacy and communication and reflection about it, and using that for building back better."* (DMH 13)
Disaster Preparedness Planning	Training first responders<br>Organisational support systems<br>Multiagency cooperation, networking and planning<br>Mapping of existing services<br>Vulnerability and needs assessments<br>Involving population and vulnerable groups in preparedness planning<br>Adapting to needs of vulnerable groups (in evacuation plans, alert systems, risk/crisis communication)<br>Adopting resilience building focus in prevention, preparedness, response and recovery activities	*"there is much more of a focus now on training up and educating first-line responders in terms of initial psychosocial response as well as non-psychosocial response, and so I think they can be combined to a degree"* (DMH 09)<br><br>*"Introducing a system in which resilience is supported, early detection of problems is possible, and referral when needed is implemented. Standardising the way workers are supported by their organization after experiencing shocking incidents."* (DMH 05)<br><br>*"Local health authority involvement in joint preparedness planning facilitated better linkages between psychosocial emergency support and routine healthcare"* (DMH 11)<br><br>*"One main strategy is multiagency cooperation, networking and planning. In Europe but also in the national context, more and more multiagency groups have formed that are doing the mental health in the disaster area but also in the prevention area, and they come together regularly and develop the disaster plans and do networking."* (DMH 12)<br>*"we have to have a map of all the services for mental health risks. So, in the refugee*

			crisis, we have the mapping, but the services are full, so we have to create new services. Then the other thing is the mapping of the people with disabilities and older people, if you don't map them beforehand, you forget them during evacuation." (DMH 12)<br><br>*"to focus on resilience building in prevention and preparedness, before events. We do a lot of resilience building with the helpers but also with the affected people, those to be affected. Because in many regions in Europe you know where the disaster might strike. If it's about flooding, for example, you already know the most vulnerable areas. But for population groups, this is just starting now."* (DMH 12)
Challenges to DMH_DRR Integration	Separate fields of practice	Different professional backgrounds<br>Mutual lack of awareness<br>Technical jargon on both sides<br>Lack of DMH stakeholder involvement in Sendai implementation	*"I'm kind of aware of the kinds of topics that they're looking into. And they seem to be very much focused on things like, you know, how can you build better buildings to prevent earthquakes knocking them down, and prediction of earthquake tremors and stuff like that, which is great you know and is very impressive, but I don't think there's much overlap at the moment. I think their professional backgrounds, I get the impression are rather different."* (DMH 01)<br><br>*"I don't know too much about the terminology of DMH, which is one of the fields where Sendai gives us real problems"* (DRR 07)<br><br>*"I really think it's the jargon, and the jargon on both sides."* (DMH 13)<br><br>*"I think both worlds tend to be a bit too inward looking. There's a bit of a mentality of seeking advice from people who you know, and who are going to think in a fairly similar sort of way. So, thinking outside of the box is important."* (DMH 09)
	Sendai vagueness	Not specifying mental health risks to be addressed<br>Broad aims could lead to all kinds of initiatives that are not the most beneficial	*"I think those sorts of very general aims within the Sendai framework are useful, but they are not very specific, and I think a lot of countries might just sort of start all sorts of activities which are not necessarily the most valuable ones"* (DMH 01)
	DMH challenges	Lacking evidence for specific psychosocial strategies<br>(Informing policy requires evidence)<br>Targeting of population-based prevention initiatives<br>Unique national systems integration	*"One of the things that I'm really concerned about is that things like Psychological First Aid, there's no evidence to prove that it works. And we've been using it for too long without getting the evidence behind all this. And if we really want the scientists to buy in, we have to have it much more evidence based. It's got to be properly evaluated. … And we've got to change how mental health sees itself, it's got to be much more evidence-based so that it makes a difference."* (DRR 01)<br><br>*"And I think there's bits of evidence that would suggest that we probably shouldn't be doing certain things as well [debriefing]. Sadly, these things don't really help the field because they're very interesting debates [debriefing debates], but they get conflated into 'counselling doesn't work' or 'psychological intervention doesn't work'. You have to be*

		really, really careful in the way these things are portrayed." (DMH 09)<br><br>*"I think these sort of general guidelines, the problem is that each country will have its own unique sort of mental health system, say in different professions, different organisational structures. So those, they have to get together to work out what their responses are going to be, and how they are going to coordinate their response within the different framework provided by each country. So, you can't, you couldn't use a UK model, so it might not work in France at all. So, I think there are general principles, and questions and issues that every country has to address, but they might go about them in different ways. But at the moment, we're not, we don't seem to be even spelling out what the problems are likely to be that the different countries should address."* (DMH 01)
Role and resource implications	Willingness to step outside one's brief and open up to challenge<br>Perception that extra work or resources are required<br>Community empowerment focus could result in less funding for 'core-work'<br>Sustainability of project-based initiatives<br>Recognising DMH role in disaster planning (not just response/recovery)	*"That's always a challenge, people's awareness, and their willingness to step outside their brief, and their ways of thinking. Because you open yourself up to being challenged, in ways that I actually take to be quite stimulating and interesting. But if you had a job in this, if you were the local authority's planner, would you necessarily welcome that challenge? I don't know, some will, some won't. And that takes you very rapidly into workforce issues. Do we have enough staff to do this, have we got the money to support it all? I'm cash strapped, they're trying to take money off me. You are asking me to do more, how do I do that. There are resource issues which are critical here, or people's perception of the resource issues."* (DMH 02)<br><br>*"There's this inherent tension between the role of a civil defence association in responding to a natural disaster in a country and having as its mandate to protect the population. But at the same time, there is a lot of work that could be done in actually educating communities to manage or mitigate their own risks as much as possible, without having to have state authority step in. And I think the tension exists, because if you come from more of an empowerment perspective, educating the community, which everyone in general thinks is a great idea, but then it means there is a decrease in funding. Because the government says, ok, we're educating a population here to do something, so what's actually going to be your role, you are not going to be the experts."* (DMH 14)<br><br>*"the most important challenge I view is that if a disaster strikes, everybody sees the importance of mental health professionals. But if you don't have a disaster, nobody sees that. So, the key players are different in the response phase and in the preparation phase. And the key players in the preparedness or prevention phase often don't see a need for mental health professionals in preparedness. They often don't see the need to bring them in beforehand. They are always seen as part of the response and recovery phase, but not of the prevention phase."* (DMH 12)

	DMH paradigm shift	Expert-based → participative approach<br>Clinical → psychosocial / needs oriented approach<br>Homogenous → differentiated population view<br>Monitoring risks in response/recovery → enhancing resilience in prevention and preparedness / assessing vulnerabilities/reducing risks beforehand	*"In Europe we always have this 'expert view' on the disaster, also in the population. So, when disaster strikes, we sit at home and wait until the expert comes and saves us. And this has to change. One strategy that has evolved is spontaneous volunteering. Ten or twenty years ago, we didn't want spontaneous volunteers, so we sent them away, but today we don't send them away anymore. And in the refugee crisis you saw very much how important this population involvement was. But we have to find good structures to bring in those volunteers and to support them."* (DMH 12)<br><br>*"I think particularly within Europe it's quite difficult for national authorities to accept that people have a certain amount of agency and empowerment. And then it very much I think again depends upon the culture of the country."* (DMH 14)<br><br>*"in civil protection and crisis management you still often find the view that 'the population' is a homogenous mass, which is behaving uniformly, and which somehow needs to be managed. So, there is still a lot of education required here, also in working with science, to better understand the population, and to bring about a paradigm shift towards a more citizen-centric crisis management."* (DMH 10)
DMH_DRR Integration opportunities	Integration avenues	Using intersectoral platforms<br>Joint stakeholder meetings, preparedness summits<br>Information and referral websites<br>Mobile phone applications<br>Screeners focussed on complaints and resources<br>Inform decision making through systematic health data<br>DRR-DMH fact sheets or DRR primer for mental health<br>Building on existing EU projects and networks to develop a shared position<br>Disasters as progress catalysts (opportunities to find new/different ways of doing things)	*"I think at some point you need to have a mental health meeting, bringing in disaster risk reduction people. … I went to a wonderful preparedness summit in the States, and they had a lot of mental health in there. … By bringing these fora to make them much more active and to get mental health on the agenda is very important. I think we have to find novel ways of doing it."* (DRR 01)<br><br>*"Most important was a national consensus process that brought together forty to fifty different stakeholder groups twice a year to develop national guidelines"* (DMH 11)<br><br>*"I think the most important thing is to use the existing networks to really meet regularly, to have this European view. Many of the disasters we have experienced in the last years didn't only happen to one nation, they were overlapping. And the EU projects have been very important in enhancing this networking at EU level."* (DMH 12)<br><br>*"The information and referral website we developed for bereaved family after the MH17 crash. Its main function was to provide accurate information, and to facilitate contact between bereaved family. I think websites such as these are a practical implementation, but also providing screeners that not only look into the level of complaints, but also the level of resources victims have should be explored further, not only for screening purposes, but also to provide feedback and information back to people"* (DMH 05)<br><br>*"information availability, interoperability, use and analysis to improve decision*

		making in the use of very complex health information. There are some organisations that already do that, but they are not too much joining forces with the typical disaster people. So, I don't see that in the national focal point for instance." (DRR 07)<br><br>*"what would be good is to write a layman's guide to the jargon in the field… or a primer on DRR for mental health folk, because we just don't know enough"* (DMH 13)<br><br>*"I think disasters can be very positive, in that one respect, in the sense of bringing people together, so that their rivalries diminish, and they will work together, for a period of time."* (DMH 02)
DMH input	Including mental health issues in disaster prevention and response planning<br>Integrating mental health knowledge at higher strategy and local planning levels<br>Provide guidance on where money, efforts and time are best spent<br>Creating the evidence base for DMH<br>Marketing DMH knowledge so it can be utilised<br>Psychosocial capacity analysis at municipal level<br>Mapping existing vulnerabilities<br>Knowledge/skill building in vulnerable groups<br>Promoting self-help approaches<br>Providing clear and understandable messages	*"having mental health professionals around the table for local disaster planning meetings"* (DMH 09)<br><br>*"include mental health professionals in prevention and preparedness to use existing knowledge on resilience building, risk communication, and risk awareness in disaster risk reduction. To include mental health professionals in planning to enhance the quality of approach in response and recovery"* (DMH 12)<br><br>*"one would hope that disaster mental health would provide a sort of very clear guidance about where money and efforts and time should best be spent."* (DMH 01)<br><br>*"that's a key challenge to get this not just to the specialists and handful of other people in this country. No, it needs to be widespread. I think we now know enough to market this. So how do we do it, how do we get other people to pick it up and run with it, on a more local basis."* (DMH 02)<br><br>*"awareness raising in stakeholders and policy makers about vulnerable groups and their needs, as well as knowledge and skill building in the population and in vulnerable groups. Risk and needs assessments are general principles in disaster mental health because they help build efficacy, reduce fears, enhance social support and mental health and wellbeing"* (DMH 12)<br><br>*"and being more proactive in communicating, and making sure, talking from a mental health point of view, that we are giving out messages that are understandable to other people, and that we are not talking in some slightly obscure language"* (DMH 09)
DRR mandate	Expanding the DRR remit to include mental health<br>Addressing psychological stressors within crisis communication<br>Incorporating mental health messaging in non-formal	*"reduction of disaster related mental health risks is an integral part of disaster risk reduction"* (DMH 12)<br><br>*"I think if you have a higher awareness of risks, it's better for your mental health*

	education / schooling<br>Expanding authorities' understanding of vulnerability (to include mental health)<br>Equipping first responders to deal with mental health problems (and manage own stress reactions)<br>Professional selection and preparation	*afterwards. Then you can mentally set and prepare yourself. You can handle things better when you know what you're up against."* (DMH 04)<br><br>*"public communication in risk reduction helps you for individual preparation, because you are aware of risks"* (DMH 03)<br><br>*"my colleagues say, communication is disaster management, good communication is good disaster management"* (DRR 04)<br><br>*"we looked at creating courses for training mental health practitioners to work with community leaders, to try and instil that kind of thinking at that level and therefore challenge some of the big blocks"* (DMH 02)
Conceptual linkages	Focus on resilience building<br>Use of health promotion strategies<br>Community-based approaches<br>IASC guideline links to community mobilization<br>Health literacy and disaster literacy<br>Healthy Cities - Urban Resilience frameworks	*"in the focus on resilience building and community approaches"* (DMH 12)<br><br>*"DRR can benefit from health promotion. We can strengthen mental health in the context of such events by incorporating existing strategies and capacities from the field of health psychology, including notions of capacity building and sustainability.* (DMH 11)<br><br>*"if you look in the IASC guidelines at action sheet 5.1, which is about mobilising communities to reflect on what they can be doing, and not even mobilising them, but putting them in a position to mobilise themselves… so this nudging described there that way of thinking is also reflected in community level DRR"* (DMH 13)<br><br>*"I think we have to start bottom up, and then 'Healthy cities' and 'Urban resilience' frameworks can provide a shared basis to view and establish this link at a local level"* (DMH 11)
Formal integration	Operationalise DMH_DRR linkages<br>DMH guidelines as Sendai appendix<br>Test effectiveness of integration and interventions<br>Put money to it (project funding)<br>Government mandating inter-professional, inter-agency planning / working groups	*"operationalising and putting money to it… a call for proposal for integrating DMH in DRR would come up with a lot of good ideas. If you then put monitoring and evaluation with it, you can put the concept to the test, so it's not just theory. So, showing that it works. I think data in the long run would make a big difference"* (DMH 13)<br><br>*"I would follow the guidelines that we've got, with a few tweaks to them… and then incorporate those plans as an appendix to the overarching thing, and then having a bit more detail to make sure there is a seamless join up between the mental health and the other elements"* (DMH 09)<br><br>*"definitely, interprofessional, interagency, intergroup working. And I think that needs*

	to be mandated from high up really." (DMH 09)

Note. 'DMH'—Disaster Mental Health; 'DRR'—Disaster Risk Reduction; 'EU'—European Union; 'IASC Guidelines'—Inter-Agency Standing Committee Guidelines on Mental Health and Psychosocial Support in Emergency Settings; 'PTSD'—Posttraumatic Stress Disorder; 'SGBV'—Sexual and gender-based violence; (Interviewee ID).
